# Etanercept biosimilars

**DOI:** 10.1007/s00296-014-3080-5

**Published:** 2014-07-01

**Authors:** Valderilio F. Azevedo, Nathalia Galli, Alais Kleinfelder, Julia D’Ippolito, Paulo C. M. Urbano

**Affiliations:** 1 Internal Medicine, Universidade Federal do Paraná, Rua Alvaro Alvin 224 casa 18, Curitiba, Paraná 80440080 Brazil; 2 Rheumatology Service, Hospital de Clínicas de Curitiba, Curitiba, Paraná Brazil; 3 Department of Clinical Trial, Edumed-Health Biotechnology, Curitiba, Paraná Brazil

**Keywords:** Etanercept, Biosimilars, TNF-α, Rheumatoid arthritis, Spondyloarthritis, Comparability

## Abstract

Etanercept was the first tumour necrosis factor alpha antagonist approved in the USA for the treatment of rheumatoid arthritis, in 1998, and then for other diseases. With the etanercept patent set to expire in the EU in 2015, a number of etanercept copies have reached the production phase and are undergoing clinical trials, with the promise of being cheaper alternatives to the reference product. In a global scenario that is favourable to the entry of biosimilars, this article discusses the stage of development, manufacture, clinical trials and the regulatory process involved in the approval of etanercept biosimilars, compiling the literature data. Reducing treatment cost is the principal attraction for biosimilars to emerge in the global market. It is essential for the doctors’ decision on the prescription of these medications, as well as for payers, to have clearly defined studies of clinical equivalence, quality, and safety in order to better evaluate the various copies of etanercept. The authors discuss the need to harmonize different national regulations and the introduction of effective pharmacosurveillance systems for prompt recognition of adverse effects in copies of biopharmaceuticals that differ from those found in the reference products.

## Introduction

The treatment of rheumatoid arthritis (RA) and other forms of spondyloarthritis was revolutionized at the end of the 1990s, after the emergence of the so-called tumour necrosis factor alpha antagonists (anti-TNFα), which are biodrugs, also known as biopharmaceuticals, produced using biotechnology [1]. These molecules are fusion proteins and monoclonal antibodies (chimeric and humanized) that specifically block the cytokine TNF-α, and in some cases, the TNF transmembrane receptor (rTNF), reducing the chronic inflammatory process [1]. The first biopharmaceutical approved in the USA for the treatment of RA, in 1998, was etanercept (a fusion protein) [2, 3]. With the near-term expiration of patents covering biologics, pharmaceutical companies around the world are developing biosimilars, which in theory are not identical, but are similar to the original biologic in terms of their protein structure, efficacy, and safety. In the USA, a key etanercept patent was due to expire in 2012, but a new patent was issued that expires in November 2028 [4, 5]. However, in the EU, the patent is set to expire in 2015 [6].

In this context, this article analyses the current situation of the principal molecules likely to be biosimilar to etanercept, and discusses the production, clinical studies, and regulation of these molecules, which face the difficult challenge of demonstrating similar efficacy and safety to the innovative molecule, with lower treatment cost.

## Etanercept

Etanercept (Enbrel^®^ Amgen-Pfizer) is a fusion protein consisting of a recombinant human TNF receptor (rTNF-p75), bound to the Fc portion of an immunoglobulin, which binds strongly to soluble TNF-α (sTNF-α) [7, 8]. It has considerable molecular weight of approximately 150,000 Da [7, 8]. Etanercept is currently used in the treatment of RA, psoriatic arthritis (PsA), ankylosing spondylitis (AS), chronic plaque psoriasis (Ps), and juvenile idiopathic arthritis (JIA) [9–11].

### Pre-clinical trials

TNFα, a macrophage-produced cytokine known for its central role in the inflammatory response and in autoimmune diseases like RA, was discovered in 1975 by the researcher Lloyd J. Old. This study enabled anti-TNF-α biopharmaceuticals to be developed soon after [12].

With the need to assess the effects of TNF-α and etanercept, it was necessary to carry out in vitro and in vivo pre-clinical trials. Although significant differences exist between disease models in animals and humans, animal in vivo studies allowed for closer study of physiological aspects, such as cell signalling, that cannot be mimicked in vitro. Type II collagen-induced arthritis (CIA) in mice, which presents similarities with RA in humans, was the initial model used to test the benefits of etanercept in vivo. Several groups carried out studies based on this model between 1991 and 1993, and all demonstrated a clear etanercept benefit for CIA, even when the treatment had begun following disease onset [13].

Two pre-clinical trials proved the efficacy of anti-TNF-α agents. The first demonstrated that an etanercept infusion in the initial stage of CIA in mice was capable of preventing destruction of the joint and improving the symptoms [14, 15]. In the second trial, also in a murine model, in which over-expression of the modified human TNF-α gene to prevent degradation of its mRNA is associated with the development of RA 4–6 months after the birth of the animals, the administration of anti-TNF-α demonstrated the prevention of arthritis [16].

### Clinical trials and main indications

#### Rheumatoid arthritis

Etanercept in association with methotrexate (MTX) is indicated for the treatment of moderate-to-severe active RA in adults, when the response to disease-modifying antirheumatic drugs (DMARDs), including MTX, was inadequate [17, 18].

The effectiveness of etanercept was assessed in a Phase II, double-blind, randomized, placebo-controlled clinical trial [19]. In this trial, 234 patients with active RA with treatment failure to at least one and not more than four DMARDs were evaluated. Doses of 10 or 25 mg of etanercept or placebo were administered subcutaneously, twice a week, for 6 consecutive months. At 3 months, 62 % of the patients treated with 25 mg of etanercept and 23 % of the patients who received placebo achieved a 20 % response as per American College of Rheumatology criteria (ACR20). At 6 months, 59 % of the group receiving 25 mg etanercept and only 11 % of the placebo group achieved ACR20. Respectively, 40 and 5 % achieved ACR50 and approximately 15 % and just over 5 % achieved ACR70 at 6 months. In this study, patients treated with etanercept achieved better outcomes in terms of disease activity and quality of life. It was also found that the therapeutic response is dose-dependent, as the results with 10-mg etanercept were intermediate to those obtained with placebo and etanercept [19].

#### Psoriatic arthritis and psoriasis

The efficacy of etanercept in the treatment of PsA was demonstrated in a placebo-controlled randomized clinical trial, in which 60 patients with active PsA that was not responsive to non-steroidal anti-inflammatory drugs (NSAIDs) received etanercept at a dose of 25 mg subcutaneously twice a week, or placebo [20]. In each group, 47 % of patients continued to use MTX at a dose of up to 25 mg/week. At week 12, 87 % of patients treated with etanercept achieved the Psoriatic Arthritis Response Criterion (PsARC) compared with 23 % of the placebo group. Furthermore, in 77 % of patients using etanercept, the response was obtained in 4 weeks or less. Similar responses were observed using ACR20, ACR50, and ACR70 criteria. Etanercept was also effective in improving skin lesions, as assessed by Psoriasis Area and Severity Index (PASI) and through the clinical improvement of active lesions identified individually [20].

The optimal dosage was evaluated by the Psoriasis Randomized Etanercept STudy in Subjects with Psoriatic Arthritis (PRESTA) clinical trial, which compared the efficacy of two therapeutic regimens of etanercept (50 mg twice weekly or 50 mg once weekly) in patients with Ps and PsA. This study revealed that higher doses of etanercept are related to better clinical outcomes in relation to skin lesions in week 12. However, both regimens obtained significant improvement in skin lesions and other aspects, such as arthritis, enthesitis, and dactylitis, at week 24 [21].

Concomitant use of MXT for the treatment of Ps and PsA is allowed, but in studies involving the five anti-TNF-α available on the market (adalimumab, etanercept, and infliximab, golimumab and certolizumab pegol), there appears to be no difference in clinical or imaging response with respect to PsA [22, 23].

#### Ankylosing spondylitis

*Etanercept is indicated* for the treatment of adults with severe active AS that is unresponsive to conventional therapy [17, 24–26].

The efficacy of etanercept in AS was observed through a double-blind, placebo-controlled randomized trial involving 277 patients with active disease, who received 25 mg of etanercept or placebo twice weekly for 24 weeks [25]. Outcome measures were Assessments in Ankylosing Spondylitis 20 % response (ASAS20) and the percentage of patients achieving higher ASAS. The measures were assessed at 2, 4, 8, 12, and 24 weeks. The outcomes showed high efficacy of etanercept at 12 weeks, and the ASAS20 was achieved by 82 (59 %) of the 138 patients in the etanercept group and by 39 (28 %) of the 139 patients in the placebo group. At 24 weeks, the ASAS20 was achieved by 79 (57 %) in the etanercept group and by 31 (22 %) patients in the placebo group. The difference between groups was significant as early as 2 weeks and was maintained over the 24-week study duration. According to Davis and collaborate, the etanercept group had significantly greater improvements in all individual components of the ASAS response criteria at weeks 12 and 24. Adverse events occurred in similar proportions of patients in each treatment group during the study.

#### Juvenile idiopathic arthritis

The most common childhood chronic rheumatic disease is JIA [27–29]. For the treatment of this disease, etanercept was used for patients aged 4–17 years who did not respond to one or more DMARDs [30].

Lovell et al. [31] conducted the first randomized, controlled study, demonstrating the safety and efficacy of etanercept in the treatment of polyarticular JIA. After that, non-controlled prospective trials were carried out, which corroborated the efficacy of etanercept in the treatment of polyarticular JIA, shown by Lovell [32–36].

The safety and efficacy of etanercept in specific categories of JIA, such as extended oligoarticular JIA (eoJIA), enthesitis-related arthritis (ERA), and PsA have not yet been completely elucidated [37–39]. Therefore, the Phase IIIb open-label, multicenter CLinical Study In Paediatric Patients of Etanercept for Treatment of ERA, PsA, and Extended Oligoarthritis (CLIPPER) trial was designed, which is currently in progress. The first part of the follow-up, conducted over 1 year, showed that treatment with etanercept 0.8 mg/kg once weekly is safe and effective for paediatric patients with eoJIA, ERA, and PsA [40].

### Pharmacodynamics (PD)

The rTNF-p75 of etanercept specifically blocks sTNF and lymphotoxin α (TNFβ), promoting lowering of serum levels of this cytokine and resulting in reduction of the inflammatory process [41, 42]. There are also reports of binding with transmembrane TNF (tTNF) [43]. The fragment, crystallizable (Fc)-fusion region in rTNF, normally used to activate the complement system in immunoglobulin G (IgG), did not demonstrate this action in etanercept [44].

Research has also demonstrated that etanercept can make cells more susceptible to apoptosis through a still unknown mechanism, in experimental colitis [45], RA [46], and in vitro studies with macrophages [47]; however, these effects did not demonstrate a clinical impact on RA or any other disease [48].

### Pharmacokinetics (PK)

Neonatal Fc receptors (FcRn) play an important role in the rescue of IgG, through their presence in the endocytic pathway in endothelial cells [49]. When IgG is internalized by means of pinocytosis, Fc receptors bind to IgG and prevent its degradation in the endosomal acid, recycling it to the cell surface, releasing it at the basic pH of blood, and thereby preventing it from undergoing lysosomal degradation [50]. This mechanism may explain the longer half-life of IgG in the blood, compared with other Isotopes. It has been demonstrated that combining certain drugs with the Fc domain of IgG significantly increases its half-life [50]. It is for this reason that etanercept is a fusion protein of TNF and Fc.

Etanercept absorption is initiated at the site of subcutaneous injection, with time to peak concentration of around 48–60 h, and elimination from the body occurs slowly with a terminal half-life between 70 and 100 h [51].

The pharmacokinetic parameters of etanercept predict that a dose of 0.8 mg/kg once weekly will generate systemic exposure comparable to 0.4 mg/kg twice weekly. Dose adjustment of etanercept is considered only when administered in conjunction with warfarin, digoxin, or MTX [51].

### Immunogenicity

The immunogenicity caused by the production of anti-drug antibodies (ADA) is also an important factor, and in many cases, an effect that prompts discontinuation of treatment [52, 53]. After long periods of treatment, ADA have been detected by the immunoenzymatic essay (ELISA). ADA may cause neutralization of the molecule, affecting PD and PK, making the treatment ineffective [54]. However, etanercept was presented as the molecule with the lowest ADA among all other anti-TNFs, but the answers about it’s low-immunogenicity still unclear [52, 54, 55].

The causes of immunogenicity can be chimeric biological drugs (e.g. infliximab), even humanized molecules (e.g. adalimumab) and fully humanized biological drugs (golimumab)—most the cases the residual immunogenicity resides in the CDR regions [56]—glycosylation profiles, fermentation, purification, formulation (aggregate formation), administration mode (i.m., i.v. and s.c.), dosing, degradation products and contaminants [57].

### Pharmacosurveillance and safety

Since the approval of etanercept (Enbrel^®^ Amgen-Pfizer) in 1998 by medicine regulatory agencies such as the EMA (European Medicines Agency) and FDA (US Food and Drug Administration), constant updates on its benefits, risks, efficacy, and safety have been necessary, a role exercised by pharmacosurveillance [58, 59]. In nearly more than 20 years of marketing, adverse effects were found such as persistent diseases (tuberculosis, hepatitis, and other infections) and cancer [52, 60–63].

The pharmacosurveillance plan includes retrospective and prospective controlled clinical trials with long-term follow-up and adverse-event reports. This information, after being collected and revised, is made available to doctors and patients by means of scientific articles and letters to the health agents, in addition to updating of the package product information leaflet with new side effects, formulation, and dose, based on the studies [64]. Another objective of the pharmacosurveillance program of the FDA is education and communication to the community. The first was created by the Immunex Corporation to facilitate self-reporting of adverse events, and the other is the healthcare program ENLIVEN, which is responsible for providing educational information and support services, as well as updates on the medicine to etanercept users, eight times a year [58].

The pharmacosurveillance study conducted by the FDA and EMA provided recognition of severities such as infections and sepsis—particularly in immunosuppressed patients who used etanercept, and the reactivation and worsening of symptoms of tuberculosis and hepatitis B [58]. The risk of reactivation of latent tuberculosis is inherent to the use of all anti-TNF agents, but etanercept appears to present lower risks, compared with other biopharmaceuticals [65]. Other side effects of note include lymphoproliferative disorders, skin cancer, haematological reactions such as thrombocytopenia, pancytopenia, and neurological disorders ranging from headache to more severe demyelinating diseases, and congestive heart failure, for example, have all been seen in patients with AS [65].

All this knowledge and updating allow new formulations and new behaviours in relation to etanercept, such as detection of tuberculosis prior to its use, and monitoring and suppression of the medication in the event of infection and immunosuppression [66].

## Manufacture of biosimilars

The production of biosimilars follows a similar process as the original biopharmaceuticals. The processing of molecules such as insulin, somatropin, interferons, and antagonists, including etanercept, starts with the use of mammalian cell lineages for replication of recombinant DNA to obtain the desired protein [67]. There are various means of synthesis available for therapeutic proteins, such as plant cells, yeasts, and bacteria (in particular *Escherichia coli*). However, the choice of mammalian cells, particularly Chinese hamster ovaries (CHOs) or murine lymphoid cells, for the production of etanercept and biosimilars, is necessary for post-translational changes and glycosylation patterns of Fc and rTNF portions, which are similar to those of human cells [68].

After stabilization of the master cell lineage, production of the molecule of interest begins. The molecule goes through a series of fermentation processes, scaling (upstream process) purification (downstream process), the pharmaceutical formulation, and finally bottling of the biopharmaceutical [69]. Besides innate growth deviations, any change in these manufacturing steps will lead to variability of the biosimilar molecule, and may affect its efficacy and safety, hence the need for clinical studies [70, 71].

The pharmaceutical formulation strategy is a critical step and needs to be accurate. The knowledge about physical and biological properties of the biological drug orientate the formulation process. Important components of protein formulations are pH, stabilizer, solubilizer, buffer, and tonicity modifier (bulking agent). The typical stability problems observed in protein pharmaceuticals are non-covalent aggregation, covalent aggregation, deamidation, cyclic imide, and cleavages [72]. This process can affect directly the efficacy (e.g. immunogenicity) and safety (e.g. adverse events) of a biological drug.

As a result, following the recommendations of the EMA and FDA, studies have used modern techniques of mass spectrometry for the analysis of etanercept ‘intended copy’ produced in China [71, 73]. Identification of the primary amino acid sequence of biosimilars, comparison of both parts of the protein separately and their glycosylation patterns, among other characteristics, have shown differences between the biosimilars manufactured. However, even biosimilars that differed from etanercept presented equivalent bioactivity [71]. This highlights an issue that has yet to be resolved by the emerging biopharmaceutical industry. The non-existence of a protocol of standardized procedures for the manufacture of biosimilars, and the lack of a sharing of know-how on new successful processes between companies [69], have hindered the establishment of a number of analytical methods for comparison between biosimilars and their reference products, or between batches already manufactured that could be considered sufficiently safe [73, 74]. Thus, the only way to ensure the safety and efficacy of the biosimilars manufactured is through conducting pre-clinical studies and clinical trials, and implementing effective pharmacosurveillance plans [74].

## Regulation

The regulation for biosimilar medicines has evolved over the past 10 years. In 2005, the EMA Committee for Medicinal Products for Human Use (CHMP), which is responsible for the scientific evaluation of human medicines authorized and marketed in Europe, published their first regulatory guideline, highlighting the required data for the licensing application for a biosimilar agent [5]. In May 2012, the guideline was published for approval of biosimilars containing monoclonal antibodies, which came into effect on 1 December 2012 [75].

Prior to 2010, the FDA had limited authority to approve biosimilars, resulting in delays in the development of these agents in the US, compared with Europe. With the promulgation of the Patient Protection and Affordable Care Act (the health reform), all this has changed. In March 2010, the law known as the Biologics Price Competition and Innovation Act (BPCIA) created a shortened approval route for biopharmaceuticals that show high similarity or interchangeability with the already-licensed biological product [76]. The law grants a 12-year exclusivity period for the manufacturer of an innovative biopharmaceutical, during which a given biosimilar product cannot be approved [77]. To stimulate the development of biosimilars, the BPCIA guarantees 1 year of exclusivity of marketing rights to the first biosimilar that is approved as being interchangeable with the reference product. In February 2012, the FDA published three preliminary documents on the development of biosimilar products to assist industry in the development of these in the USA [78–80].

The World Health Organization (WHO) guidelines on the approval of biosimilars share the same principles as the FDA and EMA guidelines in relation to the requirement of comparative data on chemistry/manufacture, pre-clinical studies, and Phase I–III clinical trials [81]. However, the regulatory environment around the world is extremely variable. In Latin America, countries tend to follow the WHO guidelines, but despite advances in the legislation, there is no harmonization of the regulations and many copy products have been approved without adequate evaluation, lacking in particular, in good-quality clinical trials. Unfortunately so far, two copies of etanercept already marketed in Mexico and Columbia cannot be considered biosimilars [81]. The full exercise of biocomparability requires the evaluation of various issues, in order for a molecule to be considered biosimilar. Issues such as analytical procedures and aspects of manufacturing of biological products are crucial for the analysis of biocomparability. These characteristics will therefore be precursors to finding the most appropriate and most sensitive study model for the evaluation of clinical outcomes that can assess the comparability of the biosimilar to the reference molecule. According to the EMA guideline, the dosage and route of administration of the biosimilar should obey the same criteria as those used by the reference molecule [77].

Another problem that will certainly affect the marketing of biosimilars is interchangeability, defined as the ability of two products to be exchanged with each other without risk of significant adverse effects on the patient’s health [82, 83]. Such interchangeability or interpermutability that is already a standard procedure for small molecules (generics) has prompted intense debate for the application in the case of copies of biopharmaceuticals. Whether a biosimilar needs to be interchangeable with a reference product, and the requirements for this procedure are issues that are still under much clinical and regulatory debate. The acceptance of interchangeability may vary from country to country, and there are several wider implications for patients, prescribers, and health systems. We believe that regulatory bodies should have transparent processes that give peace of mind to all those involved and maintain scientific standards of the choice for interchangeability of the highest level and rigour. In Europe, for example, replacing a reference product with a biosimilar is the national responsibility of each country [84]. In practice, replacement with a biopharmaceutical is not permitted in any European country [85], and it is not recommended by WHO or medical societies [86, 87]. A further issue for which there is no global standardization is the extrapolation of indications between diseases of different etiologies (neoplastic disease versus inflammatory disease). Among anti-TNFs, for example, although the therapeutic target of the different molecules are the same, TNF-α, different modes of action are demonstrable in diseases in which anti-TNFs are effective: PsA, RA, and Crohn’s disease.

## Biosimilars of etanercept

### Clinical considerations for the definition and use of biosimilars

**Fig. 1:**
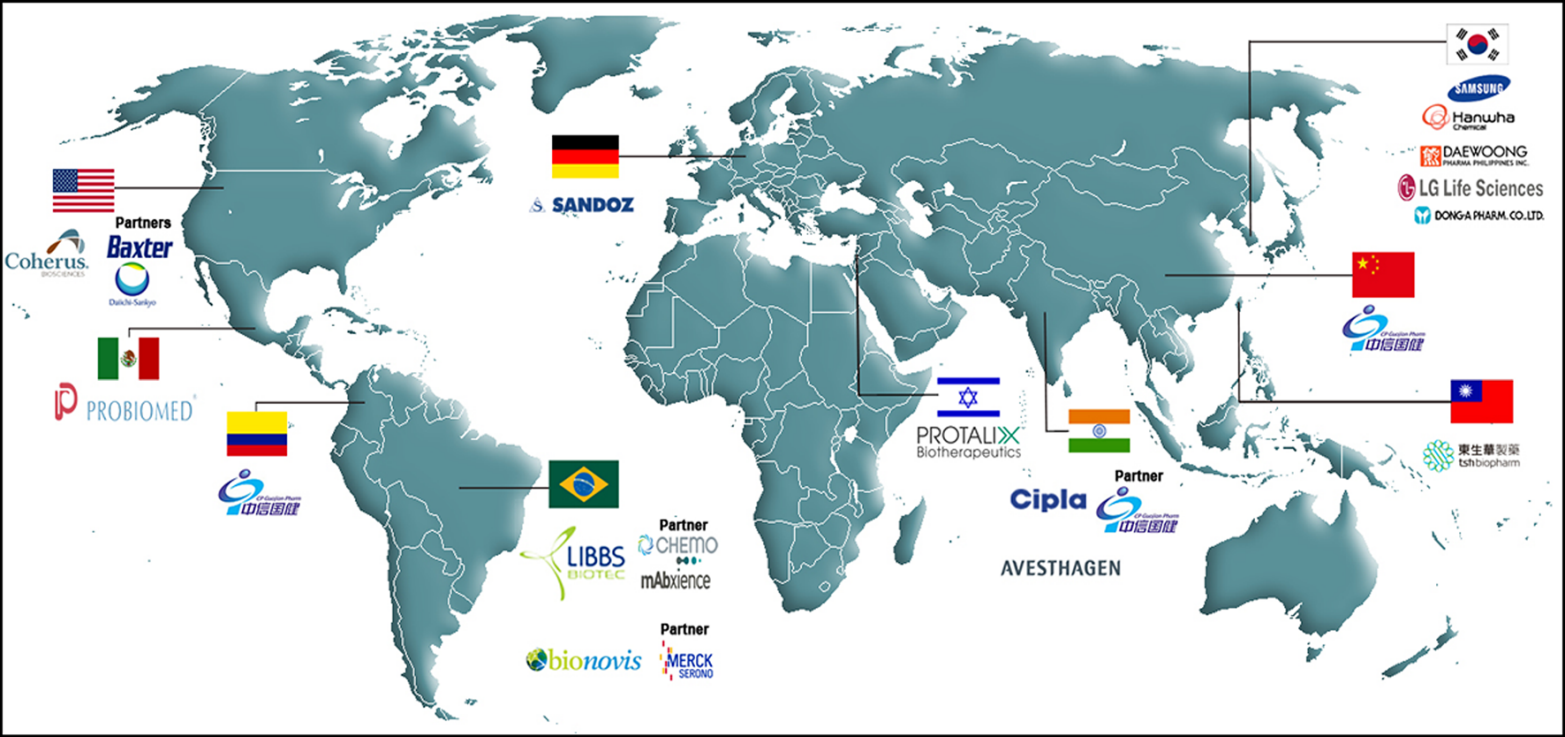
Profile of companies that are developing biosimilars or ‘intended copies’ around the world

It is well known that the demand of companies interested in producing biosimilars of etanercept has attracted the world’s attention (Fig. 1). Synthetic molecules have a simple and replicable molecular structure, enabling the production of so-called generics, which are identical copies of the original molecules [82]. Unlike chemical medicines, biopharmaceuticals have a highly complex protein structure and manufacturing process, making it impossible to produce identical copies. The commonly used term biosimilar is a regulatory definition that ultimately defines a molecule that is similar in terms of structure, efficacy, and safety in relation to the reference biopharmaceutical. Therefore, biosimilars require specific adjustment for their production and marketing [83, 88].

In order to be approved, biosimilars must go through a series of studies that include quality testing, pre-clinical and clinical trials demonstrating tolerance, pharmacokinetics, and pharmacodynamics similar to the reference biopharmaceutical product [88]. The biosimilars studied also require Phase III clinical trials, which may be of equivalence or non-inferiority in relation to the original molecule. If the comparison fails at any stage, the product cannot be elected as a biosimilar, and the term intended copy is often applied to products with incomplete biocomparability exercises [83, 89].

### Biosimilars in the clinical trial phase

There are few results published in databases from clinical trials on biosimilars of etanercept currently in development. In relation to studies found in the databases, we found the HD203, an etanercept biosimilar candidate developed by the South Korean company Hanwha Chemical, which is in a Phase I clinical trial. Yi et al. [90] carried out a double-blind, randomized, single-dose, two-sequence, and crossover study in 37 volunteers, which satisfied the bioequivalence criteria. However, the immunogenicity of HD203 was not evaluated in these studies and will therefore require further studies (e.g. studies with multiple doses).

Gu et al. [91] conducted a Phase I, single-dose, open-label, crossover trial in two sequences, involving 21 healthy male Korean volunteers. The study compared the pharmacokinetic properties of etanercept and its potential biosimilar TuNEX^®^, produced by the Taiwanese company TSH Biopharm Corp. This clinical trial showed that the biosimilar was well tolerated and met the bioequivalence criteria established by the South Korean authorities. All adverse effects reported were moderate, the most common ones being headache, inflammation of the throat, and epistaxis. However, this study had some limitations as it was a single-dose, open-label trial involving a small number of volunteers. New studies are therefore needed to access the tolerability, pharmacodynamics, and efficacy of TuNEX^®^ in patients.

In Brazil, the largest market in Latin America, Bionovis (a joint venture between EMS, Aché, Hypermarcas, and União Química) has received approval from the Brazilian National Health Surveillance Agency (ANVISA) to conduct a clinical trial with 318 patients, to evaluate the efficacy of an etanercept biosimilar molecule [92]. According to the information portal of the Ministry of Health of the Brazilian government, Bionovis formed a partnership with Merck Serono and, in addition to the etanercept biosimilar, it will produce a further six biosimilar molecules. However, there is no further information regarding pre-clinical and clinical trials or the manufacturing process of the molecule [92, 93].

Information on other biosimilars under development is registered with NIH (US National Institutes of Health) at Clinicaltrials.gov. To date, no results from these studies have been published in databases (Table 1).

**Table 1:** Profile of clinical trials on etanercept biosimilars according to international regulations

Sponsor	Biosimilar	Condition	Phase	Estimated recruitment	Experimental design	Dose (mg)	Parameter	References
Samsung Bioepis	SB4	RA (−+)	III	498	SB4 versus etanercept	50	S, E	NIH [113]
Samsung Bioepis	SB4	Healthy	I	138	SB4 versus etanercept (EU) and etanercept (US)	50	PK, S, I	NIH [114]
Sandoz	GP2015	Ps	III	372	GP2015 versus etanercept	50	E	NIH [115]
TSH Biopharm Corporation	TuNEX^®^/ENIA11	RA, MX	III	129	TuNEX^®^ versus MTX and Placebo	15–25	S, E, I	Gu et al. [91], Chen et al. [116]
LG Life Sciences	LBEC0101	Healthy	I	36	LBEC0101 versus etanercept	25	PK	NIH [117]
Daewoong Pharmaceutical	DWP422	RA	I	38	DWP422 versus etanercept	25	PK, S	NIH [118]
Hanwha Chemical	HD203	Healthy	I	44	HD203 versus etanercept	25	PK	[90]
Coherus Biosciences Inc/Baxter and Daiichi Sankyo	CHS-0214	Healthy	I	–	CHS-0214 versus etanercept	–	PK	Yi et al. [119]
Bionovis (Merck Serono)	–	–	–	318	unknown versus etanercept	–	E	Scaramuzzo [92], Brazilian Ministry of Health [93]

*Pk* pharmacokinetic, *MTX* methotrexate, *RA (*−+*)* moderate and severe rheumatoid arthritis, *S* safety, *E* efficacy, *I* immunogenicity

### Possible biosimilars candidate in the pre-clinical trial phase

Various biopharmaceuticals with similarity to etanercept are in the pre-clinical phase, particularly with a view to molecular characterization and evaluation, using in vitro and in vivo models. PRX-106 is another possible biosimilar that is being developed by the Israeli company Protalix Biotherapeutics and is in the pre-clinical phase. This molecule is produced from plant cell cultures [77, 94].

In Brazil, Libbs, in partnership with the Argentinian group Chemo, has also announced the production of a candidate etanercept biosimilar [95]. The partnership also includes mAbxience, a Swiss-based biotechnology company that belongs to Chemo Group that specializes in the development and manufacture of biosimilars [95]. According to the mAbxience website, the molecule is currently in the process of production scale-up, and pre-clinical trials will be conducted soon [96].

The Indian company Avesthagen has conducted pre-clinical trials on AVG01 (AVENT™). The molecule demonstrated high structural and pre-clinical similarity with etanercept [97]; however, there is a need for clinical trials to compare efficacy and safety in humans. The manufacture of biosimilar molecules is expanding rapidly in India, but without rigorous clinical trials, these molecules will be limited to their local market or to countries with limited regulation on the use of biopharmaceutical copies for which there are no comparative clinical trials [97].

The Korean company D-Pharm Ltd. has conducted pre-clinical trials on a possible etanercept biosimilar. In these studies, the molecule, denominated TNFR-hyFc, showed high similarity with etanercept in terms of its glycoprotein profile and pharmacokinetics [98, 99].

### Intended copies of etanercept

The term ‘intended copy’ or ‘non-comparable biologic’ differs from the definition of a biosimilar in that it lacks a complete biocomparability study and/or clinical trials, or else only limited clinical trials were conducted on it, and thus become copies that do not present similar safety and efficacy to the innovative product [100, 101]. However, some biologicals have been marketed without clinical trials in countries with less strict regulation [82]. Many intended copies cannot be recognized as biosimilars, as they do not have any studies registered with ClinicalTrials.gov. However, in some cases, the trials, although registered, were not actually carried out, and in others, there is no scientifically reliable available data in indexed journals. For the development of this article, it was sometimes necessary to use data from websites of companies that in many cases we considered inaccurate or non-transparent. This led to a limited analysis for classification of the described products.

Yisaipu, from Shanghai CP Goujian Pharmaceutical Co., is a fusion protein that has already been marketed in the Chinese market. However, data are needed on its non-inferiority in relation to etanercept to determine its biosimilarity [77, 102]. In Colombia, Yisaipu is marketed under the brand name Etanar^®^ and is not considered a biosimilar, but an intended copy, as it has only one limited clinical trial, which was not an equivalence study with the product in question [103].

In India, the company Cipla is marketing an intended copy, which according to the terms of the partnership, will be produced by the Chinese company Shanghai CP Goujian Pharmaceutical Co., the same company that produces Yisaipu. This molecule also lacks data based on the international legislation for it to be accredited as a biosimilar molecule [104].

Another country that is marketing an intended copy is Mexico, with Probiomed selling a biopharmaceutical under the brand name of Infinitam^®^. There has only been one study (unpublished), which assessed the efficacy and safety of this biopharmaceutical associated with MTX versus etanercept associated with MTX in patients with moderate and severe RA, however, is not a head-to-head comparison [105].

## Expert opinion

The globalized introduction of biopharmaceuticals has revolutionized the treatment of various diseases for which there was no treatment or the traditional treatments were ineffective or unsafe. Anti-TNF agents, for example, provide great benefit to patients with RA, spondyloarthritis, Ps, and intestinal inflammatory diseases. However, treatment with this class of products when compared with traditional molecules is more costly from a pharmacoeconomic point of view. Therefore, access to these medicines is still limited, as they cause a great impact on health budgets of various countries. With the failure of patent applications of some innovative biopharmaceuticals, a pathway has been cleared for the production of their copies. Thus, the potential cost reduction of treatment with biopharmaceuticals is, in our view, the biggest attraction for the emergence of so-called biosimilar molecules on the global market.

Due to intrinsic complexity in copying biopharmaceuticals, based on the understanding that two lines of cell production are different (reference product and copy) and usually developed independently, copies cannot be considered identical. This is recognized by various regulatory agencies, and the European regulatory authorities have, over the past 10 years, carefully established the term biosimilar, recognizing the fact that although similar to the reference products, they are not absolutely identical [106, 107]. The extensive manufacturing and clinical data available to the innovator molecule is proprietary, and includes specific details for cell line development and genetic construct, raw materials, cell culture conditions, purification parameters as well as formulation and drug delivery. These details are therefore not available to the manufacturers of any potential biosimilar product, which presents as a “knowledge gap” [108]. Due to the complexity of biological systems, and the nature of biotechnological manufacturing, any attempt to copy an originator molecule cannot result in an identical product. For this reason, providing sufficient comparability and clinical data can be provided, after regulatory approval, such follow-on biologics are termed “biosimilar”. Follow-on biologics which have not obtained regulatory approval are not regarded as biosimilars.

Etanercept is an attractive molecule when it comes to the production of copies, due to its proven efficacy, consolidated market, and high cost [82]. Even so and despite the loss of the etanercept patent outside the USA, our date survey revealed very few companies capable of manufacturing and marketing etanercept copies. Many of them, having already obtained a copy through processes of genetic reengineering, are committed to conducting the entire comparability exercise with the reference product.

It is also of critical importance to demonstrate biochemical comparability for large, complex biomolecules, such as etanercept, that the necessary detailed structural biochemical and in vitro characterization studies are completed which may potentially impact the potency, clearance, and safety, or immunogenicity profile of such biologics are executed. A pre-requisite for each process modifications for the manufacture of biologicals is that extensive biochemical characterization analyses are performed to demonstrate comparability with the product from the previously licensed process.

Such comparability evaluations need to be executed in accordance with ICH Guidelines for the assessment of Comparability of Biotechnological/Biological Products [109] and are subsequently subject to the appropriate regulatory review and approval processes. Supplemental to these data are also the comprehensive detailed, extensive set of patient safety data from pharmacovigilance programs executed throughout the development and commercial history.

Unfortunately, a few companies, particularly in Asia and Latin America, are marketing their products without any head-to-head comparison trials with etanercept [77, 81, 102–105]. Consequently, finding data on the safety and efficacy of these copies is very difficult. Thus, a comprehensive understanding of these products represents a great challenge for clinical practice and for researchers.

The current scenario of the production of etanercept copies is very heterogeneous. There are molecules whose pre-clinical development phase has been completed successfully, and molecules whose Phase I or Phase III clinical trials are in progress. It is therefore expected that marketing approval for some of these products will be obtained in the coming years by the regulatory agencies, which have rigorous and specific legislation for biosimilars. It is vital that any biosimilar (to etanercept or other biologic) will meet the same levels of biochemical, in vitro characterization, and in vivo safety and efficacy as have been demonstrated for the innovator biologic. Such product comparability and clinical data are paramount to ensuring patient safety. What is also most notable from a number of recent publications are the specific omissions in the scope of the analyses performed for key product quality attributes, which highlights the previously described “knowledge gap” [110].

Production of biosimilars is further complicated through the ‘knowledge gap’, as subtle changes to production conditions such as temperature or pH can have a profound effect on the properties of a large protein molecule, such as the extent of protein folding, glycosylation pattern or degree of aggregation. Product quality parameters such as protein glycosylation can in turn influence the therapeutic effect and safety profile of the biologic and need to be thoroughly assessed on a case–case basis [111]. For these reasons, the need for clinical trials in order to demonstrate the safety and efficacy profiles of biosimilars for each indication has been identified [108, 112]. This position precludes interchangeability by a pharmacist between a biosimilar and the originator’s product.

Another notable scenario in countries like Brazil, attracted our attention, wherein certain manufacturers of biosimilars have formed partnerships with local national companies for the development of their products. In this partnership, production technology is transferred with the guarantee that in the future, the government will prioritize the purchase of these products over those of other manufacturers, for a period of up to 5 years [93].

It is essential for doctors’ decision-making on the prescription of etanercept biosimilars, as well as for payers, to have well-characterized studies of equivalence or non-inferiority, in order to ensure accurate assessment of their copies. The aims of these comparative clinical trials are to collect initial data for follow-up and to assess uncertainties related to the safety and efficacy of a biosimilar compared with the innovative product. Certainly, regulations have advanced in the sense of guaranteeing scientific rigour in the process of approval of copies, and for this reason, the term biosimilar has been widely used for products that are proven to have physical and chemical similarity and also similarity based on pre-clinical and clinical trials. Despite this, we found some inconsistencies in which, despite the advanced and rigorous legislation on the approval of biosimilars, as in the case of Mexico, two products that were copies of etanercept had received approval without the verification of equivalence trials to determine comparability with the reference product [81, 105].

At the national and global level, it is essential to ensure harmonization of the different regulations for the approval of biosimilars, particularly in relation to the introduction of effective pharmacosurveillance systems for easier recognition of adverse effects of copies of biological medicines that are different from those already found in the reference products. Pharmacosurveillance requires sufficient identification of a suspect agent of an adverse event; thus, it is essential for health professionals to be accurate in the identification of a product during the process of communicating (reporting) an adverse event. Biosimilars are a challenge in this regard, because the identification of the active substance [through an International Nonproprietary Name (INN)] does not provide a sufficient description to differentiate possible safety problems between the biosimilar and the reference product, in the process of manufacture and handling. For this, we believe that biosimilars must be quickly identified as distinct medicines, with different INN/USAN. The brand name, for example, should also be used to ensure specificity, within an accurate regulatory system. When there is no clarity in this distinction, there is a high likelihood that this will hinder the identification of an agent suspected of causing a serious adverse event.

In case the originally authorized medicinal product has more than one indication, and this is the case for antiTNF agents, the efficacy and safety of the medicinal product claimed to be similar has to be justified or, if necessary, demonstrated separately for each of the claimed indications. In certain cases, it may be possible for regulatory authorities to extrapolate therapeutic similarity shown in one indication to other indications of the reference medicinal product. The Justification will depend on clinical experience, available literature data, whether or not the same mechanisms of action or the same receptor(s) are involved in all indications. Some concerns have been raised with the extrapolation of antiTNFs because of the different mode of actions in different diseases like RA and psoriasis (although the therapeutic targets are the same).

Finally, it makes good sense that the pharmacosurveillance requirements for biosimilars be as rigorous as those required for the reference products.

To monitor adverse effects and promote long-term safety for patients, as has already occurred with etanercept, all of its potential biosimilars should have post-marketing requirements, risk managements plans, and other pharmacosurveillance protocols, aligned with those required by etanercept. When it comes to biosimilars, everything is changing quickly: regulations, new manufacturers, and health policies, making this a very dynamic environment. This article, based on scientific information and the indexed literature, is an attempt to collate the various experiments currently in progress with etanercept copies. We also take this opportunity, as clinicians, to emphasize the aspects related to the pharmacosurveillance of biological products. We recognize that the global scenario will probably change considerably with the presence of new players in the coming years, which will certainly require a new revision of this article.

## Abbreviations

ACR: American College of Rheumatology
ADA: Anti-drug antibodies
ANVISA: Brazilian National Health Surveillance Agency
AS: Ankylosing spondylitis
ASAS: Assessments in Ankylosing Spondylitis
BPCIA: Biologics Price Competition and Innovation Act
CHMP: Committee for Medicinal Products for Human Use
CHO: Chinese hamster ovary
CIA: Collagen-induced arthritis
DMARD: Disease-modifying antirheumatic drug
ELISA: Enzyme-linked immunosorbent assay
EMA: European Medicines Agency
ERA: Enthesitis-related arthritis
eoJIA: Early onset juvenile idiopathic arthritis
Fc: Fragment crystallizable
FDA: Food and Drug Administration
IgG: Immunoglobulin G
INN: International Nonproprietary Name
JIA: Juvenile idiopathic arthritis
MTX: Methotrexate
NIH: National Institute of Health
NSAID: Non-steroidal anti-inflammatory drug
PASI: Psoriasis Area and Severity Index
PD: Pharmacodynamics
PK: Pharmacokinetic
PRESTA: Psoriasis Randomized Etanercept Study in Subjects with Psoriatic Arthritis
Ps: Plaque psoriasis
PsA: Psoriatic arthritis
PsARC: Psoriatic Arthritis Response Criterion
RA: Rheumatoid arthritis
TNF: Tumour necrosis factor
sTNF: Soluble tumour necrosis factor
tTNF: Transmembrane tumour necrosis factor
rTNF: Tumour necrosis factor receptor
USAN: United States Adopted Names
WHO: World Health Organisation
